# Mental health, potential minority stressors and resilience: evidence from a cross-sectional survey of gay, bisexual and other men who have sex with men within the Celtic nations

**DOI:** 10.1186/s12889-021-12030-x

**Published:** 2021-11-06

**Authors:** Arlene McGarty, Lisa McDaid, Paul Flowers, Julie Riddell, John Pachankis, Jamie Frankis

**Affiliations:** 1 Institute of Health & Wellbeing, University of Glasgow, 1st floor Admin Building, Gartnavel Royal Hospital, 1055 Great Western Road, G12 0XH, Glasgow, Scotland UK; 2 Institute for Social Science Research, The University of Queensland, Long Pocket Precinct, 80 Meiers Rd, Indooroopilly, Brisbane, QLD 4068 Australia; 3 MRC/CSO Social and Public Health Sciences Unit, University of Glasgow, Top Floor, 200 Renfield Street, G2 3AX, Glasgow, Scotland UK; 4 School of Psychological Sciences & Health, University of Strathclyde, 40 George Street, G1 1QE, Glasgow, UK; 5 Yale School of Public Health, 60 College St, New Haven, CT USA; 6 School of Health and Life Sciences, Glasgow Caledonian University, Cowcaddens Road G4 0BA, Glasgow, Scotland UK

**Keywords:** Men who have sex with men, Minority stressors, Resilience, Mental health, Depression, Anxiety

## Abstract

**Background:**

Gay, bisexual and other men who have sex with men (GBMSM) are at a greater risk of mental health problems, such as anxiety and depression, than heterosexual adults. Numerous factors and stressors have been reported to impact men’s mental health, although it has been suggested that resilience could have a protective effect. The aim of this study is to explore mental health, minority stressors, and resilience among a large online cross-sectional survey of GBMSM in the Celtic nations.

**Methods:**

Data for this cross-sectional study were collected from the Social Media, GBMSM and Sexual and Holistic Health (SMMASH2) self-report online survey. Participants (*n* = 3077) were recruited via gay sociosexual media in Scotland, Wales, Northern Ireland, and the Republic of Ireland. Binary logistic regression analyses were conducted to identify factors that increased the odds of moderate-to-severe anxiety and depression. Potentially relevant variables (*p* < 0.05) were carried forward in hierarchal logistic regression analyses.

**Results:**

The prevalence of moderate-to-severe anxiety and depression was 19.9 and 14.4%, respectively. Having a disability (OR = 1.73) and having financial worries sometimes/all of the time (OR = 1.93) increased the odds of having moderate-to-severe depression and anxiety, respectively. No minority stressors were associated with depression, whereas experiencing any form of relationship abuse in the last 12 months significantly increased the odds of anxiety (OR = 1.50). Resilience, namely a sense of coherence, had a protective effect and significantly reduced the odds of moderate-to-severe depression (OR = 0.85) and anxiety (OR = 0.89).

**Conclusions:**

Disability and financial worries were associated with increased depression and anxiety, respectively, while resilience had a protective effect for GBMSM in the SMMASH2 study. Future research is needed to better understand the role of resilience and the challenges and stresses of everyday life and intersecting health problems. Future research is also needed that incorporates the perspectives of those most affected by mental ill-health to co-develop effective solutions that respond to their contextual surroundings.

## Background

Mental health is a core component of overall health [1]. However, the prevalence of mental ill-health varies across the population, with sexual minorities, such as gay, bisexual and other men who have sex with men (GBMSM), at a greater risk of increased mental health problems than heterosexual men. The risk of depression and anxiety is 1.5 times higher in sexual minorities, compared to heterosexuals [2], with rates of depressive symptoms and disorders between sexual minorities and heterosexuals greater during adolescence (OR = 2.94) [3]. In the United Kingdom (UK), GBMSM have a higher prevalence of ill-health, including depression and substance use, and are more likely to report risky sexual behaviours than heterosexual men [4]. Depression and anxiety are common in GBMSM (21.3 and 17.1%, respectively), with age, education level, and income influencing mental health [5].

Factors impacting the mental health of GBMSM are varied, with numerous stressors, e.g. stigma, discrimination, social stress and exclusion, heterosexism, and the experience or threat of violence, promoting feelings of helplessness and hopelessness that can develop into depression and suicidality [2, 3, 6–9]. This aligns with the Minority Stress Theory, which suggests that socially determined stressors, such as discrimination and victimisation, inhere to being part of a minority group and can impact mental health [3]. Non-disclosure of sexual identity can also result in an increased risk of mental health problems, with levels of ‘outness’ to others impacting the association between syndemic conditions and sexual risk taking [10, 11].

Resilience refers to positive adaptation, whereby a person has or maintains positive mental health despite experiencing adversity [12, 13]. Various factors have been suggested as impacting resilience in GBMSM. For example, ‘outness’ may result in GBMSM experiencing less stress due to increased positive social support and stronger connections to the gay community, which could be associated with more resilience to negative or chaotic life experiences [10]. However, resilience is a complex construct and social phenomena, with theories of resilience highlighting that varying life circumstances can mean that individuals in a shared risk group (e.g. GBMSM) may not experience comparable levels of risk. For example, GBMSM who have strong and positive social networks and/or family relationships that report better mental health are not necessarily more resilient than GBMSM who report lower mental health, but instead they may face lower proximal risk due to their strong networks [14]. This relates to the construct of sense of coherence, which argues that the way a person views their life (i.e. as comprehensible, manageable, and meaningful) can impact their health, with a higher sense of coherence protecting against negative aspects of life [15–17]. Emotional competency is a further important aspect of resilience, which describes the positive response to emotion of oneself and others, with higher emotional competency demonstrating higher resilience [18].

The relationship between mental health, minority stress, and resilience among GBMSM has not been examined in the UK or Irish context. Despite the evidence base demonstrating poor mental health among GBMSM, mental health interventions with sexual minorities are limited [19]. There is a lack of evidence-based interventions that specifically target sexual minority mental health, particularly in adults, with there still being many unknowns regarding prevention and treatment [3]. In addition, most of the existing evidence-base is North American, with limited UK and European studies [7]. This paper develops the existing evidence base by exploring mental health, minority stressors, and resilience among a large online cross-sectional survey of GBMSM in the Celtic nations - Scotland, Wales, Northern Ireland, and the Republic of Ireland - to inform future policy and intervention development. The present study aimed to investigate:

1. The prevalence and association of depression and anxiety symptoms with sociodemographic, behavioral, and stress factors; and
2. The impact of minority stressors and resilience on depression and anxiety levels.

## Methods

### Design

Data for this cross-sectional study were collected from The Social Media, MSM and Sexual and Holistic Health (SMMASH2) self-report online survey, administered between April and June 2016. Ethical approval was granted by Glasgow Caledonian University School of Health and Life Sciences Ethics Subcommittee (HLS id: HLS/NCH/15/26).

### Population and recruitment

Adult men who identified as gay, bisexual, or sought sex with other men were recruited through gay-specific sociosexual media websites (Gaydar, Squirt, and Recon) and smartphone applications (apps; Hornet, Gaydar, Grindr, Growlr, and Recon). Men were eligible to participate if their profile location, computer IP address, or smartphone GPS coordinates were located in Scotland, Wales, the Republic of Ireland, or Northern Ireland. Recruitment involved sending each eligible profile two messages inviting participation in the survey, as well as banner advertisements on the participating websites/smartphone apps. The link in the messages and banners took participants to the survey webpage that provided full details of the survey and specified that survey completion was taken as consent to participate.

No power calculations were conducted as it is not possible to generate representative samples of GBMSM and therefore researchers use different sampling frames theorised to be representative of sub-sections of the population, i.e. online surveys of sociosexual media. Our data are broadly representative of men who use sociosexual media to seek male sex partners. This gives greater access to men who do not live in large cities, but is limited by issues of digital poverty, literacy and access, and well as social desirability related to online samples of sexual behaviours.

### Measures

#### Predictor variables

The self-report questionnaire developed by Frankis, Flowers and McDaid [20] surveyed sociodemographic [age, country, ethnicity, sexual orientation, education, employment status, relationship status, disability or long term condition (excluding mental health for the purpose of these analyses), financial worries, proximity to the gay scene, frequency of gay scene use, frequency of gay social media website / app use, and HIV-status] and behavioural variables [high risk condomless anal sex (CAI; defined here as reporting CAI with > = 2 partners or with casual/HIV-status unknown/serodiscordant partners in the last year. This measure does not adjust for either PrEP or TasP, though in 2016 PrEP use was very low in these Celtic countries.

The questionnaire developed by Frankis et al. [20] also assessed hazardous alcohol use (using the Fast Alcohol Screening Tool), party drug use (defined here as using speed, crystal methamphetamine, cocaine, ecstasy, GHB/GBL, ketamine, or mephedrone in the last year) and chemsex (defined here as reporting sex while using at least one of the 4 main UK chemsex drugs: crystal methamphetamine, mephedrone, GHB/GBL or ketamine in the last year).

Minority stress was measured as gay-related stigma, reported ‘outness’ and any reported relationship abuse in the past year. The Gay-Related Stigma Scale, a 20-item adaptation of the HIV stigma scale [21, 22], measures personalised stigma and concealment stigma (range 0–80), with higher scores suggesting higher gay-related stigma. This scale was reviewed by lay GBMSM and sexual health experts in the Celtic Nations. All items were retained following small changes for cultural appropriateness resulting in good face validity. A single item assessed ‘outness’ from 1 (not out to anyone) to 5 (out to everyone) [20].

Relationship abuse was measured using a modified version of the Sex and Relationships Problems Scale [23], modified for GBMSM in the Celtic nations, following review by lay GBMSM and sexual health experts, which investigated experiences of sexual, physical, and emotional abuse from a partner or ex-partner in the previous 12 months [20].

Resilience was measured as sense of coherence and emotional competency using the 13-item Sense of Coherence – Orientation to Life questionnaire, a reliable (Cronbach’s α = 0.88) measure of sense of coherence, based on Antonovsky’s [16] 29-item scale [20], validated for use in the Celtic Nations [24]. It measures resilience to stressful life situations that may negatively impact health (range 0–78), with higher scores indicating higher levels of sense of coherence.

Emotional competency was measured using the 30-item Trait Emotional Intelligence Questionnaire, which is a reliable (Cronbach’s α = 0.93) measure of a person’s ability to understand and regulate emotions to improve health, with scores ranging from 1 (low emotional competency) to 7 (high emotional competency) [18, 20], validated for use in the Celtic Nations [25]. For each scale, higher scores indicated higher resilience.

#### Outcome variables

The nine-item Patient Health Questionnaire-9 (PHQ-9) [26] measures depression-related feelings and problems (e.g. low mood, sleep problems, or changes in appetite) experienced in the previous 2 weeks, which has been validated for use in adults in the Celtic nations [27]. Results are scored between 0 (not affected by any issue at all) and 27 (affected by every issue nearly every day), with depression severity categorised as: none (score 0–4), mild (score 5–9), moderate (score 10–14), moderately severe (score 15–19) or severe (score 20–27).

Anxiety was measured using the seven-item Generalized Anxiety Disorder-7 questionnaire (GAD-7) [28]. This tool measures anxiety-related feelings and problems (e.g. feeling nervous, trouble relaxing, or irritable) experienced over the previous 2 weeks, with results categorised as: none (score 0–4), mild (score 5–9), moderate (score 10–14), or severe (score 15–21) anxiety [29]. Although these are not diagnostic tools, both are frequently used to assess current depression and anxiety respectively in cross-sectional studies and intervention evaluations and are validated for use in the Celtic Nations [29].

#### Analysis

A total of *n* = 4690 GBMSM completed the questionnaire. Data from participants not living in the Celtic nations (*n* = 767) and those missing over 75% of data on the variables listed in Table 1 (*n* = 706) were excluded from the analysis. This resulted in an overall sample size of *n* = 3077.

**Table 1:** Demographic variables

Variable		N	Percentage (%)
**Mental health**			
Anxiety	None/Mild (≤14)	1829	80.1%
	Moderate/Severe (> 14)	454	19.9%
Depression	None/Mild (< 10)	1985	85.6%
	Moderate/Severe (≥10)	334	14.4%
**Sociodemographic**			
Age	18–25	538	17.5%
	26–35	713	23.2%
	36–45	695	22.6%
	46+	1124	36.6%
Gender identity	Transgender	191	6.3%
	Not transgender	2846	93.7%
Country	Scotland	1474	47.9%
	Wales	491	16.0%
	Northern Ireland	232	7.5%
	Republic of Ireland	880	28.6%
Ethnicity	White	2974	97.0%
	Black	5	0.2%
	Asian	38	1.2%
	Mixed / other	48	1.6%
Sexual Orientation	Gay	2443	80.1%
	Bisexual	575	18.8%
	Straight/other	33	1.1%
Educated post-secondary	No	1165	38.9%
	Yes	1831	61.1%
Employment	Employed/ self employed	2137	73.9%
	Other (including retired, carer, student)	755	26.1%
Relationship status	Single	1869	61.0%
	Regular Male Partner	643	21.0%
	Civil Partnership / Married Man	247	10.0%
	Regular Female Partner	306	10.0%
Any Disability	No	1848	67.6%
	Yes	887	32.4%
Any financial worries	Occasionally/Never	1659	57.0%
	Sometimes - All of the time	1249	43.0%
Nearest gay venue with easy reach	Too far	494	17.0%
	4	319	11.0%
	3	515	17.8%
	2	670	23.11%
	Nearby	659	22.7%
	Don’t know	242	8.3%
HIV status	HIV positive	222	8.3%
	HIV negative	2100	78.1%
	Untested	367	13.6%
**Behavioural**			
Frequency of gay website use	At least once a day	959	41.4%
	Every few days / weekly	764	33.0%
	Monthly or less	274	11.8%
	Never / stopped using	319	13.8%
Frequency of gay App use	At least once a day	1107	48.0%
	Every few days / weekly	464	20.1%
	Monthly or less	149	6.5%
	Never / stopped using	587	25.4%
Frequency of gay scene use	4–5 times a week	23	0.8%
	2–3 times per week	335	11.5%
	1–2 times per week	145	5.0%
	Once a month or less	903	31.1%
	Never	1497	51.6%
Condomless sex	No	1734	64.1%
	Yes	972	35.9%
Alcohol use	Safe	1398	65.3%
	Hazardous	743	34.7%
Chemsex use in last 12 months	No	2035	87.6%
	Yes	289	12.4%
**Minority stressors**			
Stigma (higher score = higher stigma) 0–80	Mean (SD)	23.4 (11.2)	
Outness	Out to no one	410	14.1%
	Out to some or all	2501	85.9%
Any experience of relationship abuse in last 12 months	No 2176	1708	78.5%
	Yes	468	21.5%
**Resilience**			
Emotional competency	Mean (SD)	3.3 (0.9)	
Sense of coherence	Mean (SD)	40.4 (13.4)	

All analyses were conducted in SPSS version 24. For all analyses, the outcomes of anxiety and depression were categorised as; no/mild symptoms (GAD-7 score ≤ 14; PHQ-9 score < 10) and moderate-to-severe symptoms (GAD-7 score > 14; PHQ-9 score ≥ 10). Stigma, sense of coherence, and emotional competence were included as continuous variables with higher scores representing higher stigma, sense of coherence, and emotional competence, respectively; these have been fully described in previous Measures section. Outness was modelled as a binary variable (out to no one / out to some or all).

First, descriptive statistics were calculated for sociodemographic, behavioural, minority stressors, and resilience variables, alongside depression (PHQ-9) and anxiety (GAD-7), and all percentages were calculated based on the number of participants who responded to each variable. Next, relationships between sociodemographic and behavioural variables, and depression and anxiety (no/mild symptoms or moderate-to-severe symptoms) were investigated using binary logistic regression.

Finally, variables that were significant (*p* < 0.05) at the bivariate level were entered into a hierarchical logistic regression model used to estimate odds ratios and 95% confidence intervals for mental health outcomes. Model 1 included sociodemographic and behavioural variables; Model 2 added minority stress variables; Model 3 added resilience variables, and Model 4 added interactions between minority stressors and each of the resilience factors entered separately. Separate hierarchical logistic regression models were built for depression and anxiety. Multicollinearity was tested for each model; there were no significant correlations between explanatory variables and therefore all variables were included in the analyses.

## Results

### Sociodemographic, behavioural, and minority stress characteristics

Descriptive statistics for sociodemographic, behavioural, and minority stress characteristics are presented in Table 1. The mean age of participants was 39 years (range 16–78 years, SD = 13.5) with just under half of the sample living in Scotland (47.9%). Most (97.0%) identified as white and the majority (80.1%) identified as gay. Over half had at least post-secondary school level education (61.1%) and most were employed (73.9%). One in ten (10.0%) reported having a regular female partner, 31.0% reported a regular male partner (including civil partnerships and being married to a man) though the majority were single (61.0%). Almost one-third (32.4%) reported a disability (as defined by the UK census but excluding mental health issues) and 43.0% reported at least some financial worries. Most reported they were HIV negative (78.1%), 8.3% were HIV positive, and 13.6% said they do not know their HIV status. While our recruitment strategy meant that most participants reported frequent gay website (74.4% weekly) and app (68.1% weekly) use, 82.7% reported their frequency of gay scene use as only once a month or never. Over one third reported condomless sex in the last year (35.9%). Over one third also reported hazardous levels of drinking (34.7%) and 12.4% reported party drug use in the last 12 months. The majority of men (85.9%) were out to some or all people and 21.5% reported experience of relationship abuse in the last year. In relation to resilience, mean scores for emotional competency and sense of coherence were 3.3 (SD = 0.9) and 40.4 (SD = 13.4), respectively.

### What is the prevalence of self-reported depression and anxiety?

Overall, 19.9% (*n* = 454) were categorised as having moderate-to-severe anxiety and 14.4% (*n* = 334) as having symptoms of moderate-to-severe depression (see Table 1) in the past 2 weeks.

### What sociodemographic and behavioural factors are associated with self-reported depression and anxiety?

The results of the bivariate analysis are shown in Table 2. Eight sociodemographic factors were significant. Being aged ≤25 years, no post-secondary school education, not being employed, being single, reporting a non-mental health disability, reporting financial worries, and being HIV positive significantly increased the odds of reporting moderate-to-severe depression, whereas living in the Republic of Ireland significantly reduced the odds. Four behavioural factors (more frequent gay sociosexual media app use, reporting condomless anal sex, problematic alcohol use, and using party drugs in last 12 months) significantly increased the odds of reporting moderate-to-severe depression.

**Table 2:** Bivariate associations with depression and anxiety

Predictor variables		Depression				Anxiety			
		N	Odds ratio	95% CI	***p***-value	N	Odds ratio	95% CI	p-value
**Sociodemographic factors**									
Age (years)* †	18–25	408	REF	REF	REF	395	REF	REF	REF
	26–35	506	0.79	0.57, 1.11	0.17	497	0.71	0.52, 0.96	0.03
	36–45	546	0.67	0.48, 0.95	0.02	542	0.63	0.47, 0.86	0.00
	46+	855	0.46	0.33, 0.64	0.00	844	0.38	0.28, 0.51	0.00
Country*	Scotland	1126	REF	REF	REF	1112	REF	REF	REF
	Wales	381	1.12	0.82, 1.54	0.48	377	0.94	0.70, 1.25	0.66
	Northern Ireland	174	1.18	0.77, 1.81	0.45	166	0.99	0.67, 1.48	0.97
	Republic of Ireland	638	0.72	0.54, 0.97	0.03	628	0.76	0.59, 0.98	0.04
Ethnicity	White	2247	REF	REF	REF	2214	REF	REF	REF
	Other	66	0.81	0.39, 1.72	0.59	63	1.38	0.78, 2.46	0.27
Sexual Orientation†	Gay	1897	REF	REF	REF	1854	REF	REF	REF
	Bisexual	380	0.74	0.52, 1.03	0.07	388	0.70	0.52, 0.94	0.02
	Straight/other	22	0.56	0.13, 2.41	0.44	21	0.63	0.18, 2.14	0.45
Educated post- secondary*†	No	865	REF	REF	REF	853	REF	REF	REF
	Yes	1415	0.57	0.45, 0.72	0.00	1389	0.63	0.51, 0.77	0.00
Employment*†	Other (including retired, carer, student)	609	REF	REF	REF	606	REF	REF	REF
	Employed/ self employed	1691	0.44	0.34, 0.56	0.00	1659	0.46	0.37, 0.58	0.00
Relationship status*†	Single	1428	REF	REF	REF	1396	REF	REF	REF
	Regular Male Partner	480	0.52	0.37, 0.72	0.00	484	0.74	0.57, 0.96	0.02
	Civil Partnership / Married Man	191	0.48	0.29, 0.80	0.05	184	0.38	0.23, 0.62	0.00
	Regular Female Partner	210	0.33	0.19, 0.58	0.00	210	0.39	0.25, 0.61	0.00
Any Disability*†	No	1447	REF	REF	REF	1434	REF	REF	REF
	Yes	728	4.80	3.74, 6.16	0.00	709	3.57	2.87, 4.44	0.00
Any financial worries*†	Occasionally / Never	1314	REF	REF	REF	1298	REF	REF	REF
	Sometimes / All of the time	997	3.83	2.97, 4.93	0.00	976	3.58	2.87, 4.45	0.00
Nearest gay venue with easy reach	Don’t know	166	REF	REF	REF	168	REF	REF	REF
	Too far	401	0.90	0.58, 1.50	0.68	389	1.41	0.87, 2.30	0.16
	4	263	1.04	0.61, 1.79	0.88	262	1.45	0.87, 2.42	0.16
	3	414	1.03	0.62, 1.70	0.90	409	1.52	9.40, 2.45	0.09
	2	547	1.00	0.61, 1.62	0.98	538	1.31	0.82, 2.09	0.26
	Nearby	519	0.81	0.49, 1.33	0.40	506	1.31	0.82, 2.12	0.26
HIV status*†	HIV positive	193	REF	REF	REF	192	REF	REF	REF
	HIV negative	1809	0.66	0.45, 0.97	0.34	1777	0.59	0.42, 0.83	0.00
	Don’t know	304	0.99	0.62, 1.57	0.95	302	0.79	0.52, 1.20	0.27
**Behavioural factors**									
Frequency of gay scene use	Never - 4/5 times a week (1–5)	2312	0.97	0.85, 1.10	0.64	2274	0.92	0.82, 1.03	0.15
Frequency of website use	Never - Daily use (1–4)	2229	0.94	0.84, 1.05	0.29	2233	0.94	0.85, 1.04	0.23
Frequency of App use*†	Never - Daily use (1–4)	2222	1.18	1.07, 1.31	0.01	2226	1.18	1.08, 1.29	0.00
Condomless sex *	No	1450	REF	REF	REF	1432	REF	REF	REF
	Yes	842	1.31	1.03, 1.66	0.03	824	1.23	0.99, 1.51	0.06
Problematic alcohol use*†	Safe	1343	REF	REF	REF	1352	REF	REF	REF
	Hazardous	719	1.34	1.04, 1.73	0.03	716	1.38	1.11, 1.73	0.01
Party drug use in last 12 months*†	No	1933	REF	REF	REF	1898	REF	REF	REF
	Yes	386	1.40	1.05, 1.87	0.02	385	1.33	1.03, 1.73	0.03
**Minority stressors**									
Stigma* †	Low to high (0–80)	1959	1.03	1.02, 1.05	0.00	1949	1.03	1.02, 1.04	0.00
Outness* †	Not out to anyone - Out to everyone (1–5)	2314	1.15	1.06, 1.26	0.00	2278	1.15	1.07, 1.24	0.00
Experienced any form of relationship abuse in last 12 months*†	No	1611	REF	REF	REF	1589	REF	REF	REF
	Yes	445	2.69	2.07, 3.51	0.00	439	3.16	2.49, 4.02	0.00
**Resilience factors**									
Emotional competency*†	Low - High (1–7)	1810	6.86	5.42, 8.69	0.00	1816	5.11	4.21, 6.19	0.00
Sense of coherence*†	Low - High (0–13)	1835	0.86	0.85, 0.88	0.00	1837	0.87	0.86, 0.89	0.00

*Taken forward to depression hierarchal logistic regression analysis (p < 0.05)

†Taken forward to anxiety hierarchal logistic regression analysis (p < 0.05)

The hierarchical regression analyses for depression are shown in Table 3. In Model 1, three sociodemographic factors (relationship status, disability, and financial worries) were significant predictors of moderate-to-severe depression. Specifically, having any disability (excluding mental health; OR 3.3; 95% CI 2.3, 4.7) and reporting financial worries sometimes/all of the time (OR 2.8; 95% CI 2.0, 4.1) increased the odds of moderate-to-severe depression, whereas having a regular male partner significantly decreased the odds of moderate-to-severe depression (OR 0.5; 95% CI 0.3, 0.9). No behavioural factors were significant in Model 1. In the final multivariate model, when all factors and interactions were included (Model 4), reporting a disability was the only sociodemographic or behavioural factor that increased the odds of moderate-to-severe depression (OR 1.7; 95% CI 1.1, 2.7).

**Table 3:** Hierarchal binary logistic regression analysis for depression

Variable		Model 1			Model 2			Model 3			Model 4		
		**Odds ratio**	**95% CI**	**p-value**	**Odds ratio**	**95% CI**	**p-value**	**Odds ratio**	**95% CI**	**p-value**	**Odds ratio**	**95% CI**	**p-value**
**Sociodemographic factors**													
Age (years)	18–25	REF	REF	REF	REF	REF	REF	REF	REF	REF	REF	REF	REF
	26–35	1.01	0.61, 1.67	0.98	0.98	0.58, 1.65	0.94	1.03	0.57, 1.85	0.92	1.01	0.56, 1.82	0.98
	36–45	0.86	0.49, 1.51	0.59	0.75	0.42, 1.33	0.32	0.82	0.43, 1.58	0.56	0.81	0.42, 1.55	0.52
	46+	0.60	0.35, 1.02	0.06	0.57	0.33, 0.99	0.05*	0.77	0.41, 1.46	0.42	0.76	0.40, 1.45	0.41
Country	Scotland	REF	REF	REF	REF	REF	REF	REF	REF	REF	REF	REF	REF
	Wales	1.05	0.64, 1.72	0.85	0.98	0.59, 1.63	0.94	1.23	0.69, 2.19	0.48	1.23	0.69, 2.19	0.49
	Northern Ireland	0.80	0.40, 1.61	0.53	0.75	0.37, 1.52	0.42	1.08	0.50, 2.32	0.85	1.08	0.50, 2.34	0.85
	Republic of Ireland	0.77	0.50, 1.19	0.24	0.78	0.50, 1.22	0.28	0.80	0.48, 1.35	0.40	0.78	0.46, 1.33	0.36
Educated post- secondary	No	REF	REF	REF	REF	REF	REF	REF	REF	REF	REF	REF	REF
	Yes	0.76	0.53, 1.09	0.14	0.79	0.55, 1.15	0.22	0.95	0.62, 1.45	0.82	0.96	0.63, 1.47	0.86
Employment	Other (including retired, carer, student)	REF	REF	REF	REF	REF	REF	REF	REF	REF	REF	REF	REF
	Employed/ self employed	0.75	0.50, 1.12	0.16	0.78	0.51, 1.78	0.23	0.81	0.51, 1.31	0.39	0.82	0.51, 1.33	0.43
Relationship status	Single	REF	REF	REF	REF	REF	REF	REF	REF	REF	REF	REF	REF
	Regular Male Partner	0.54	0.33, 0.89	0.02*	0.53	0.32, 0.87	0.01*	0.65	0.37, 1.17	0.15	0.63	0.35, 1.13	0.12
	Civil Partnership / Married Man	0.60	0.28, 1.25	0.17	0.56	0.26, 1.20	0.13	1.12	0.46, 2.68	0.82	1.05	0.44, 2.53	0.92
	Regular Female Partner	0.53	0.22, 1.27	0.15	0.69	0.27, 1.77	0.44	0.99	0.33, 2.95	0.99	1.13	0.37, 3.45	0.83
Any Disability	No	REF	REF	REF	REF	REF	REF	REF	REF	REF	REF	REF	REF
	Yes	3.26	2.27, 4.68	0.00*	3.05	2.10, 4.43	0.00*	1.67	1.09, 2.57	0.02*	1.73	1.12, 2.66	0.01*
Any financial worries	Occasionally / Never	REF	REF	REF	REF	REF	REF	REF	REF	REF	REF	REF	REF
	Sometimes / All of the time	2.84	1.96, 4.11	0.00*	2.38	1.62, 3.48	0.00*	1.42	0.92, 2.19	0.11	1.40	0.91, 2.17	0.13
HIV status	HIV positive	REF	REF	REF	REF	REF	REF	REF	REF	REF	REF	REF	REF
	HIV negative	1.08	0.59, 1.97	0.81	1.11	0.59, 2.07	0.75	1.77	0.84, 3.72	0.14	1.78	0.84, 3.77	0.13
	Don’t know	1.23	0.59, 2.59	0.58	1.27	0.59, 2.74	0.54	1.71	0.69, 4.22	0.24	1.74	0.70, 4.30	0.23
**Behavioural factors**													
Frequency of App use	Never - Daily use (1–4)	0.99	0.84, 1.17	0.94	0.97	0.82, 1.15	0.75	1.01	0.83, 1.23	0.93	1.01	0.83, 1.23	0.94
Condomless sex	No	REF	REF	REF	REF	REF	REF	REF	REF	REF	REF	REF	REF
	Yes	1.37	0.95, 1.96	0.10	1.42	0.97, 2.06	0.07	1.11	0.73, 1.70	0.63	1.13	0.74, 1.74	0.57
Problematic alcohol use	Safe	REF	REF	REF	REF	REF	REF	REF	REF	REF	REF	REF	REF
	Hazardous	1.15	0.79, 1.67	0.47	1.19	0.81, 1.74	0.37	1.30	0.85, 1.99	0.24	1.26	0.82, 1.94	0.29
Part drug use in last 12 months	No	REF	REF	REF	REF	REF	REF	REF	REF	REF	REF	REF	REF
	Yes	1.13	0.72, 1.75	0.60	1.11	0.70, 1.76	0.65	1.30	0.78, 2.17	0.31	1.31	0.79, 2.18	0.30
**Minority stressors**													
Stigma	Low to high (0–80)				1.05	1.03, 1.07	0.00*	1.01	0.99, 1.03	0.37	1.01	0.99, 1.03	0.44
Outness	Not out to anyone - Out to everyone (1–5)				1.37	1.14, 1.65	0.00*	1.31	1.05, 1.63	0.02*	0.30	0.05, 1.79	0.19
Experienced any form of relationship abuse in last 12 months	No				REF	REF	REF	REF	REF	REF	REF	REF	REF
	Yes				1.43	0.96, 2.13	0.08	1.13	0.71, 1.80	0.60	1.15	0.72, 1.82	0.56
**Resilience factors**													
Emotional competency	Low - High (1–7)							3.02	2.06, 4.44	0.00*	1.23	0.32, 4.72	0.76
Sense of coherence	Low - High (0–13)							0.92	0.90, 0.95	0.00*	0.85	0.76, 0.94	0.00*
**Interactions**													
How out are you * Emotional Competency											1.25	0.91, 1.71	0.18
How out are you * Sense of Coherence											1.02	1.00, 1.05	0.10
Cox and Snell R square		0.11			0.14			0.26			0.27		
Nagelkerke R Square		0.20			0.24			0.47			0.47		
Percentage correct		86.00%			86.40%			89.00%			89.40%		

*Statistically significant at p < 0.05

In the bivariate analyses of sociodemographic factors associated with anxiety (see Table 2), being single, having any disability (excluding mental health), and having financial worries at least sometimes increased the odds of moderate-to-severe anxiety. Whereas being aged 18–25 years, living in the Republic of Ireland, being bisexual, educated post-secondary school, employed, and HIV negative reduced the odds of having moderate-to-severe anxiety. Problematic alcohol use and party drug use were the only significant behavioural factors.

In Model 1 of the hierarchal regression (Table 4), the odds of having moderate-to-severe anxiety were higher for men who reported having any disability (OR 2.6; 95% CI 1.9, 3.6) and financial worries sometimes/all of the time (OR 3.3; 95% CI 2.4, 4.6) and were significantly lower for men who were aged 46+ years (OR 0.5; 95% CI 0.3, 0.7), employed/self-employed (OR 0.7; 95% CI 0.5, 1.0), or married / in a civil partnership (OR 0.4; 95% CI 0.2, 0.9). No behavioural factors were statistically significant. In the final multivariate model when all variables and interactions were included (Model 4), having financial worries was the only sociodemographic or behavioural factor which increased the odds of having moderate-to-severe anxiety (OR 1.9; 95% CI 1.3, 2.8).

**Table 4:** Hierarchal binary logistic regression analysis for anxiety

Variable		Model 1			Model 2			Model 3			Model 4		
		**Odds ratio**	**95% CI**	**p-value**	**Odds ratio**	**95% CI**	**p-value**	**Odds ratio**	**95% CI**	**p-value**	**Odds ratio**	**95% CI**	**p-value**
**Sociodemographic factors**													
Age (years)	18–25	REF	REF	REF	REF	REF	REF	REF	REF	REF	REF	REF	REF
	26–35	0.81	0.51, 1.27	0.36	0.81	0.51, 1.30	0.38	0.88	0.52, 1.48	0.63	0.87	0.52, 1.47	0.61
	36–45	0.77	0.47, 1.27	0.31	0.70	0.42, 1.16	0.17	0.78	0.44, 1.36	0.38	0.77	0.44, 1.36	0.37
	46+	0.45	0.28, 0.73	0.00*	0.44	0.27, 0.72	0.00*	0.60	0.34, 1.04	0.07	0.60	0.34, 1.03	0.07
Sexual orientation	Gay	REF	REF	REF	REF	REF	REF	REF	REF	REF	REF	REF	REF
	Bisexual	0.98	0.58, 1.64	0.93	1.13	0.65, 1.97	0.67	1.09	0.59, 2.00	0.80	1.06	0.57, 1.97	0.85
	Straight /other	0.60	0.06, 6.20	0.67	0.87	0.09, 8.63	0.91	0.77	0.06, 9.29	0.84	0.76	0.06, 9.48	0.83
Educated post- secondary	No	REF	REF	REF	REF	REF	REF	REF	REF	REF	REF	REF	REF
	Yes	0.79	0.58, 1.09	0.15	0.84	0.61, 1.17	0.30	1.00	0.69, 1.44	0.99	1.00	0.69, 1.45	0.99
Employment	Other (including retired, carer, student)	REF	REF	REF	REF	REF	REF	REF	REF	REF	REF	REF	REF
	Employed/ self employed	0.68	0.47, 0.98	0.04*	0.71	0.49, 1.03	0.07	0.72	0.47, 1.10	0.13	0.72	0.48, 1.10	0.13
Relationship status	Single	REF	REF	REF	REF	REF	REF	REF	REF	REF	REF	REF	REF
	Regular Male Partner	0.77	0.51, 1.15	0.20	0.73	0.48, 1.12	0.15	0.93	0.58, 1.50	0.78	0.93	0.58, 1.49	0.75
	Civil Partnership / Married Man	0.42	0.20, 0.89	0.02*	0.42	0.19, 0.91	0.03*	0.62	0.26, 1.46	0.27	0.61	0.26, 1.44	0.26
	Regular Female Partner	0.54	0.23, 1.29	0.16	0.62	0.25, 1.52	0.29	0.93	0.34, 2.57	0.89	0.99	0.35, 2.77	0.98
Any Disability	No	REF	REF	REF	REF	REF	REF	REF	REF	REF	REF	REF	REF
	Yes	2.63	1.90, 3.63	0.00*	2.38	1.70, 3.33	0.00*	1.33	0.91, 1.95	0.14	1.35	0.92, 1.98	0.13
Any financial worries	Occasionally / Never	REF	REF	REF	REF	REF	REF	REF	REF	REF	REF	REF	REF
	Sometimes / All of the time	3.28	2.37, 4.56	0.00*	2.82	2.02, 3.96	0.00*	1.93	1.33, 2.81	0.00*	1.93	1.32, 2.80	0.00*
HIV status	HIV positive	REF	REF	REF	REF	REF	REF	REF	REF	REF	REF	REF	REF
	HIV negative	0.79	0.47, 1.36	0.40	0.85	0.49, 1.49	0.58	1.13	0.59, 2.16	0.72	1.12	0.59, 2.15	0.73
	Don’t know	0.98	0.50, 1.90	0.94	1.05	0.53, 2.10	0.89	1.22	0.55, 2.71	0.62	1.22	0.55, 2.69	0.63
**Behavioural factors**													
Frequency of App use	Never - Daily use (1–4)	1.00	0.86, 1.15	0.95	0.99	0.85, 1.15	0.91	1.00	0.84, 1.18	0.99	1.00	0.84, 1.18	0.96
Problematic alcohol use	Safe	REF	REF	REF	REF	REF	REF	REF	REF	REF	REF	REF	REF
	Hazardous	1.21	0.86, 1.69	0.27	1.27	0.90, 1.78	0.18	1.37	0.94, 2.01	0.10	1.36	0.93, 1.99	0.11
Part drug use in last 12 months	No	REF	REF	REF	REF	REF	REF	REF	REF	REF	REF	REF	REF
	Yes	1.00	0.67, 1.49	0.99	0.93	0.61, 1.41	0.74	0.90	0.57, 1.43	0.66	0.91	0.58, 1.43	0.67
**Minority stressors**													
Stigma	Low to high (0–80)				1.04	1.02, 1.06	0.00*	1.01	0.99, 1.03	0.61	1.01	0.98, 1.03	0.65
Outness	Not out to anyone - Out to everyone (1–5)				1.28	1.08, 1.52	0.01*	1.24	1.02, 1.50	0.04*	0.72	0.16, 3.15	0.66
Experienced any form of relationship abuse in last 12 months	No				REF	REF	REF	REF	REF	REF	REF	REF	REF
	Yes				1.88	1.31, 2.69	0.00*	1.50	1.00, 2.25	0.05	1.50	1.00, 2.26	0.05*
**Resilience factors**													
Emotional competency	Low - High (1–7)							2.00	1.44, 2.79	0.00*	1.52	0.53, 4.39	0.44
Sense of coherence	Low - High (0–13)							0.92	0.90, 0.94	0.00*	0.89	0.82, 0.97	0.01*
**Interactions**													
How out are you * Emotional Competency											1.07	0.83, 1.38	0.59
How out are you * Sense of Coherence											1.01	0.99, 1.03	0.41
Cox and Snell R square		0.13			0.16			0.28			0.28		
Nagelkerke R Square		0.21			0.26			0.45			0.45		
Percentage correct		82.00%			82.60%			86.50%			86.30%		

Statistical significance set at p < 0.05

### Are depression and anxiety associated with minority stressors?

At the bivariate level, all minority stressors were significant predictors of depression and anxiety (Table 2). Specifically, higher stigma, being out to more people and reporting relationship abuse increased the odds of both moderate-to-severe depression and anxiety. For depression, when minority stressors were entered into regression Model 2 (Table 3), higher levels of stigma (OR 1.1; 95% CI 1.0, 1.1) and being out to more people (OR 1.37; 95% CI 1.1, 1.7) remained significant and increased the odds of moderate-to-severe depression. However, in Model 4, when all factors and interactions were included, no minority stressors remained significant predictors of depression. For anxiety (Table 4), all minority stressors were significant in Model 2, with higher stigma (OR 1.0; 95% CI 1.0, 1.1), being out to more people (OR 1.3; 95% CI 1.1, 1.5), and reporting relationship abuse (OR 1.9; 95% CI 1.3, 2.7) increasing the odds of moderate-to-severe anxiety. In Model 4 of the hierarchal regression, having experienced abuse in a relationship was the only minority stressor that was significantly associated with anxiety, although only marginally (OR 1.5; 95% CI 1.0, 2.3; *p* = 0.0499).

### Does resilience have a protective effect on depression and anxiety?

At the bivariate level, all resilience measures were significant predictors of depression and anxiety (Table 2). Specifically, higher emotional competency and lower sense of coherence increased the odds of reporting moderate-to-severe depression and anxiety.

For depression, when resilience variables were added into regression Model 3 (Table 3), both remained significant, with higher emotional competency (OR 3.0; 95% CI 2.1, 4.4) and lower sense of coherence (OR 0.9; 95% CI 0.9, 1.0) increasing the odds of moderate-to-severe depression. In Model 4, when all factors and interactions were included in the model, sense of coherence remained the only significant resilience variable, with lower sense of coherence increasing the odds of moderate to severe depression (OR 0.9; 95% CI 0.8, 0.9). The final model provided the highest predictive power (percentage correct = 89.4%, R^2^ = 0.5; Table 3) with no statistically significant interactions.

For anxiety, both resilience factors were significant in regression Model 3, with higher emotional competency (OR 2.0; 95% CI 1.4, 2.8) and lower sense of coherence (OR 0.9; 95% CI 0.9, 0.9) increasing the odds of moderate-to-severe anxiety. In Model 4, sense of coherence remained the only significant resilience variable, with lower sense of coherence increasing the odds of anxiety (OR 0.9; 95% CI 0.8, 1.0). There were no significant interactions in this model. In the hierarchal anxiety model (Table 4), Model 3 of this regression provided a slightly higher predictive power (percentage correct = 86.50%, R^2^ = 0.5) compared to the Model 4 when interactions were added (percentage correct = 86.3%, R^2^ = 0.5).

## Discussion

This paper is the first UK study to examine the association of minority stressors and resilience with depression and anxiety among GBMSM. We found that over one in seven men reported moderate-to-severe symptoms of depression and one in five reported moderate-to-severe symptoms of anxiety. Numerous social, contextual, and behavioural factors were associated with depression and anxiety in the bivariate analyses, including age, education, employment, relationship status, disabilities (excluding mental health), any financial worries, HIV status, and hazardous alcohol use. However, having a disability and financial worries were the only significant predictors of depression and anxiety in the final hierarchical models. A similar result was found for minority stressors, with higher stigma, outness, and experiencing relationship abuse increased the odds of moderate-to-severe depression and anxiety, yet none remained significant in the final hierarchal model for depression. However, having experienced abuse in a relationship was a significant predictor of anxiety. Regarding the protective effect of resilience, the odds of moderate-to-severe depression and anxiety decreased with higher sense of coherence but *increased* with higher emotional competency in the bivariate analyses. However, only sense of coherence showed a protective effect for anxiety and depression in the final model. The implications of these findings are discussed below.

The key sociodemographic factors associated with mental ill-health were more frequent financial worries and reporting any disability (excluding mental health). Clearly, financial worries can be experienced by all, regardless of sexual orientation, but this highlights the broader structural influences on the lives of GBMSM. With the impacts of austerity and more recently COVID-19, this could highlight further exclusion of GBMSM due to service cuts, which disproportionally impact the lesbian, gay, bisexual, and transgender (LGBT) populations and can impact anxiety [30]. This could also suggest impacts of employment status on financial worries, as GBMSM still experience discrimination in the workplace, including restricted job choice and career progression [31]. The association with disability highlights the potential impact of intersectionality on the lives and mental health of GBMSM, just as it does for the general population. However, this is an under-researched area. Various factors could contribute to increased mental ill-health of people living with disabilities, such as desexualisation [32], and deserves further research.

All minority stressors were significant in the bivariate analyses and almost all minority stressors were significant in the multivariate analyses, which highlights their relevance to depression and anxiety. This corroborates previous research which has demonstrated that minority stressors increase the odds of anxiety and depression [3, 7]. However, in general, these variables were not significant in the final multivariate models, which suggests that resilience has an impact on the relationship between minority stressors and mental health. Experiencing any form of relationship abuse in the last 12 months was the only minority stressor that was significant in the final multivariate model and associated with significantly increased odds of anxiety. Experiencing relationship abuse was the only minority stressor not significant in any of the depression multivariate models, further highlighting that different factors are associated with anxiety and depression in GBMSM. These findings somewhat contrast with previous research which has demonstrated that experiencing relationship abuse, in particular physical abuse, significantly predicted depression but only marginally predicted anxiety [33]. However, as LGBT populations experience higher levels of intimate partner violence than heterosexual adults, this is an important area for future research [34].

We hypothesised that resilience would have a protective effect on anxiety and depression. Higher sense of coherence, indicative of greater resilience, was significantly associated with lower odds of depression and anxiety. However, higher emotional competency was associated with increased odds of anxiety and depression in the bivariate analyses and Model 3 of the hierarchal regression analyses. Yet, it was not statistically significant in the final model (Model 4) when interactions were included, even though no interactions were significant. This suggests the role of resilience is complex and not yet fully understood. It is possible that different aspects of resilience could influence mental health problems differently, which is an important consideration for future intervention development. For example, while high emotional competency suggests greater skill in regulating emotions, this enhanced awareness may be counter-productive to regulating mental health in the face of potentially pervasive stigma-related stressors [35]. Moreover, these results suggest that sense of coherence is a more important predictor of mental health than emotional competency. Therefore, this is an important finding in relation to future intervention development and identifying GBMSM who might benefit from intervention support. This also supports previous research that has identified an ongoing need to better understand resilience in this population [36, 37].

### Future research directions

As this is the first study to explore mental health, minority stressors, and resilience in GBMSM in the Celtic nations, additional research needs to be conducted in this population group to increase the evidence-base. Specifically, we need to better understand resilience and the underlying mechanisms of sense of coherence and emotional competence. Better understating these links and effects on anxiety and depression could have important impacts on patient care and health policy relating to the mental health of GBMSM. Future research should also incorporate the perspectives of GBMSM who are most affected by mental ill-health to co-develop effective solutions that respond to their contextual surroundings.

### Strengths and limitations

This study adds to the evidence base on the reported experience of mental health, minority stressors and resilience among GBMSM, particularly outside North America. It is the first study to investigate associations of resilience and mental health among sexual minority men in the UK context. This study also used thorough multi-step analyses, thus increasing the reliability and validity of results. Moreover, the key measures of depression and anxiety are based on recent (< 2 weeks) reports and demonstrate good clinical accuracy.

The primary weakness of this study is its reliance on self-report data from an online, non-probability sample of men. In addition, measuring recall on the previous 12 months may limit the accuracy of the data; however, there are limited methods available to measure our included variables objectively, and the increased burden of more objective measures would limit the feasibility of this design and recruitment. There may also be bias with this sample that could impact external validity; specifically, the questionnaire is long, which may impact uptake and completion rates, and participation was limited to people who had access to the internet and received the invite email. While we found few associations between minority stressors and mental health, we did not have data on all potential measures associated with Minority Stress Theory, so caution should be taken in interpreting these results. Future research is required to hypothesise, understand, and determine the role of minority stress on GBMSM in the UK Celtic nations.

## Conclusions

This research adds to the evidence base on the need for mental health interventions focussed specifically with GBMSM. It highlights that the factors associated with depression and anxiety vary. Therefore, future research needs to better understand the role of minority stressors, resilience and the challenges and stresses of everyday life; for example, how financial pressures and intersecting health problems, such as disability, impact the mental health of GBMSM in the Celtic nations. It points to a need to work with the groups most affected by mental ill-health to co-develop more effective solutions and build a better evidence base for what works, with whom, and in what context. Taking account of the context in which people live is important when considering health inequalities. This should be considered within a complex adaptive systems model when developing multi-level interventions that can take a synergistic, salutogenic effect to promote and sustain positive mental health among GBMSM [38–40].

## Data Availability

The data that support the findings of this study are available from the final author upon reasonable request, subject to agreeing to statements of intent, confidentiality and non-disclosure. The data are not publicly available nor stored in a repository since neither ethical approval nor participant consent were sought for these purposes.

## Abbreviations

Apps: applications
CI: confidence intervals
GAD-7: Generalized Anxiety Disorder-7 questionnaire
HIV: human immunodeficiency viruses
LGBT: lesbian, gay, bisexual, and transgender
GBMSM: men who have sex with men
OR: odds ratio
PHQ-9: Patient Health Questionnaire-9
SMMASH2: The Social Media, GBMSM and Sexual and Holistic Health
SD: standard deviation
UK: United Kingdom

## References

[1] WHO. Mental Health Action Plan 2013–2020. Geneva: WHO; 2013.

[2] King M, Semlyen J, Tai SS, Killaspy H, Osborn D, Popelyuk D, Nazareth I (2008). A systematic review of mental disorder, suicide, and deliberate self harm in lesbian, gay and bisexual people. BMC psychiatry.

[3] Lucassen MF, Stasiak K, Samra R, Frampton CM, Merry SN (2017). Sexual minority youth and depressive symptoms or depressive disorder: a systematic review and meta-analysis of population-based studies. Aust N Z J Psychiatry.

[4] Mercer CH, Prah P, Field N, Tanton C, Macdowall W, Clifton S, Hughes G, Nardone A, Wellings K, Johnson AM, Sonnenberg P (2016). The health and well-being of men who have sex with men (MSM) in Britain: evidence from the third National Survey of sexual attitudes and lifestyles (Natsal-3). BMC Public Health.

[5] Hickson F, Davey C, Reid D, Weatherburn P, Bourne A (2017). Mental health inequalities among gay and bisexual men in England, Scotland and Wales: a large community-based cross-sectional survey. J Public Health.

[6] Marshal MP, Dietz LJ, Friedman MS, Stall R, Smith HA, McGinley J, Thoma BC, Murray PJ, D'Augelli AR, Brent DA (2011). Suicidality and depression disparities between sexual minority and heterosexual youth: a meta-analytic review. J Adolesc Health.

[7] Plöderl M, Tremblay P (2015). Mental health of sexual minorities. A systematic review. Int Rev Psychiatry.

[8] Plöderl M, Wagenmakers EJ, Tremblay P, Ramsay R, Kralovec K, Fartacek C, Fartacek R (2013). Suicide risk and sexual orientation: a critical review. Arch Sex Behav.

[9] Pompili M, Lester D, Forte A, Seretti ME, Erbuto D, Lamis DA, Amore M, Girardi P (2014). Bisexuality and suicide: a systematic review of the current literature. J Sex Med.

[10] Pitpitan EV, Smith LR, Goodman-Meza D, Torres K, Semple SJ, Strathdee SA, Patterson TL (2016). “Outness” as a moderator of the association between syndemic conditions and HIV risk-taking behavior among men who have sex with men in Tijuana. Mexico AIDS Behav.

[11] Pachankis JE, Mahon CP, Jackson SD, Fetzner BK, Bränström R (2020). Sexual orientation concealment and mental health: a conceptual and meta-analytic review. Psycol Bull.

[12] Herrman H, Stewart DE, Diaz-Granados N, Berger EL, Jackson B, Yuen T (2011). What is resilience?. Can J Psychiatr.

[13] Wald J, Taylor S, Asmundson GJG, Jang KL, Stapleton J (2016). Literature review of concepts: psychological resilience.

[14] Luthar SS, Cicchetti D, Becker B (2000). The construct of resilience: a critical evaluation and guidelines for future work. Child Dev.

[15] Antonovsky A (1979). Health, stress and coping.

[16] Antonovsky A (1987). Unraveling the mystery of health: how people manage stress and stay well.

[17] Eriksson M, Lindström B (2007). Antonovsky’s sense of coherence scale and its relation with quality of life: a systematic review. J Epidemiol Community Health.

[18] Petrides KV, Furnham A (2003). Trait emotional intelligence: Behavioural validation in two studies of emotion recognition and reactivity to mood induction. Eur J Personal.

[19] Wang J, Häusermann M, Weiss MG (2014). Mental health literacy and the experience of depression in a community sample of gay men. J Affect Disord.

[20] Frankis J, Flowers P, Welsh M, McDaid L (2018). Low levels of chemsex among men who have sex with men, but high levels of risk among men who engage in chemsex: analysis of a cross-sectional online survey across four countries. Sex Health.

[21] Berger BE, Ferrans CE, Lashley FR (2001). Measuring stigma in people with HIV: psychometric assessment of the HIV stigma scale. Res Nurs Health.

[22] Frost DM, Parsons JT, Nanín JE (2007). Stigma, concealment and symptoms of depression as explanations for sexually transmitted infections among gay men. J Health Psychol.

[23] Bailey JV, Pavlou M, Copas A, McCarthy O, Carswell K, Rait G, Hart G, Nazareth I, Free C, French R, Murray E (2013). The Sexunzipped trial: optimizing the design of online randomized controlled trials. J Med Internet Res.

[24] Eriksson M, Lindström B (2005). Validity of Antonovsky’s sense of coherence scale: a systematic review. J Epidemiol Community Health.

[25] Andrei F, Siegling AB, Aloe AM, Baldaro B, Petrides KV (2016). The incremental validity of the trait emotional intelligence questionnaire (TEIQue): a systematic review and meta-analysis. Journal of personality assessment. J Pers Assess.

[26] Kroenke K, Spitzer RL, Williams JB (2001). The PHQ-9: validity of a brief depression severity measure. J Gen Intern Med.

[27] Levis B, Benedetti A, Thombs BD (2019). on behalf of the DEPRESsion Screening Data (DEPRESSD) Collaboration 2019. Accuracy of Patient Health Questionnaire-9 (PHQ-9) for screening to detect major depression: individual participant data meta-analysis. BMJ.

[28] Spitzer RL, Kroenke K, Williams JB, Lowe B (2006). A brief measure for assessing generalized anxiety disorder: the GAD-7. Arch Intern Med.

[29] Manea L, Gilbody S, McMillan D (2015). A diagnostic meta-analysis of the patient health Questionnaire-9 (PHQ-9) algorithm scoring method as a screen for depression. Gen Hosp Psychiatry.

[30] Mitchell M, Beniner K, Rahim N, Arthur S (2013). Implications of austerity for LGB&T People and services.

[31] Hudson-Sharp N, Metcalf H (2016). Inequality among lesbian, gay bisexual and transgender groups in the UK: a review of evidence.

[32] McRuer R, Mollow A (2012). Sex and disability.

[33] Reuter TR, Newcomb ME, Whitton SW, Mustanski B (2017). Intimate partner violence victimization in LGBT young adults: demographic differences and associations with health behaviors. Psychol Violence.

[34] Edwards KM, Sylaska KM (2015). Neal AM. Intimate partner violence among sexual minority populations: A critical review of the literature and agenda for future research Psychol Violence.

[35] Soto JA, Armenta BE, Perez CR, Zamboanga BL, Umana-Taylor AJ, Lee RM (2012). Strength in numbers? Cognitive reappraisal tendencies and psychological functioning among Latinos in the context of oppression. Cultur Divers Ethnic Minor Psychol.

[36] Colpitts E, Gahagan J (2016). The utility of resilience as a conceptual framework for understanding and measuring LGBTQ health. Int J Equity Health.

[37] de Lira AN, de Morais NA (2018). Resilience in lesbian, gay, and bisexual (LGB) populations: an integrative literature review. Sex Res Social Policy.

[38] Rutter H, Savona N, Glonti K, Bibby J, Cummins S, Finegood DT, Greaves F, Harper L, Hawe P, Moore L, Petticrew M (2017). The need for a complex systems model of evidence for public health. Lancet..

[39] Stall R, Matthews DD, Friedman MR, Kinsky S, Egan JE, Coulter RW, Blosnich JR, Markovic N (2016). The continuing development of health disparities research on lesbian, gay, bisexual, and transgender individuals. Am J Public Health.

[40] McDaid L, Flowers P, Ferlatte O, McAloney-Kocaman K, Gilbert M, Frankis J. Informing theoretical development of salutogenic, asset-based health improvement to reduce syndemic ill-health among gay, bisexual and other men who have sex with men: empirical evidence from secondary analysis of multi-national, online cross-sectional surveys. SSM Population Health 2020; 10 (available online 10.1016/j.ssmph.2019.100519). 10.1016/j.ssmph.2019.100519 PMC6911981 31853476

